# Mothers’ lived experience of caring for children with inborn errors of amino acid metabolism

**DOI:** 10.1186/s12887-023-03946-x

**Published:** 2023-06-07

**Authors:** Sara Shirdelzade, Monir Ramezani, Peyman Eshraghi, Abbas Heydari

**Affiliations:** 1 Department of Pediatrics, School of Nursing and Midwifery, Mashhad University of Medical Sciences, Mashhad, Iran; 2 Nursing and Midwifery Care Research Center, Mashhad University of Medical Sciences, Mashhad, Iran; 3 Department of Pediatric and Endocrinology, School of Medicine, Mashhad University of Medical Sciences, Mashhad, Iran

**Keywords:** Lived experience, Mother, Inborn errors of metabolism, Children, Qualitative

## Abstract

**Background:**

Inborn errors of amino acid metabolism are chronic conditions that have many sequels. Mothers of these children are facing different challenges which are underdetermined. This study was done to explore lived experience of mothers caring for these children.

**Methods:**

This is an interpretive phenomenology with van Manen’s approach which has 6 steps. Data were gathered by convenience and purposeful sampling. Nine mothers with different experiences were interviewed and the interviews were audiotaped.

**Results:**

Six final themes were revealed from the exploring mothers’ experiences including the future tied to the past, psychosis in the shadow of a lost ideal child, rebellion and blaming, the ways of escaping difficulties, self-forgetting in the shadow of full-time care, passing difficulties in the duality of hope-hopelessness, caring in a continuum of isolation-socialization.

**Conclusion:**

Mothers have multiple challenges in taking care of their children, especially psychologically and financially. So, nurses must plan programs for helping mothers of children with inborn errors of amino acid metabolism to reduce the effects of disease on mothers and consequently the children and the whole family.

**Supplementary Information:**

The online version contains supplementary material available at 10.1186/s12887-023-03946-x.

## Background

Inborn errors of amino acid metabolism (IEAAMs) are a type of Inborn Errors of Metabolism (IEMs) that significantly contribute to intellectual disability, seizures, sudden infant death syndrome (SIDS), and neurological disorders [1]. These disorders arise from defects in metabolic pathways, and the accumulation of defective pathway metabolites causes the associated signs and symptoms. These metabolites can be poisonous or cause cell destruction due to their aggregation in organelles. Additionally, the deficiency of lower-path metabolites is also involved in disease pathogenesis, which can affect one or more systems [2–4]. The signs of these disorders can iclude encephalopathy, metabolic acidosis, hyperammonemia, coma, liver damage, cataracts, kidney failure, kidney cysts, cardiomyopathy, pericarditis, and more. [2, 4, 5]. The incidence of these disorders varies by country, with a range of 1/800–2500 live births, the lowest being in Japan (10/100,000 live births) and the highest in Mexico (350/100,000 live births) [2]. Although, there is no official information on Iran, it is prevalent in other Middle Eastern countries like Saudi Arabia and Bahrain, which have cultural and social proximities with Iran (1/1443, and 1/6000 live births, respectively) [4, 6].

IEAAMs are categorized as rare and chronic conditions that require lifelong support, protection, care, and treatment [3, 7]. Having a child with such a condition is extremely difficult for mothers, as they may experience pain, feel ill, or be unable to do the things that other children can [8]. These diseases, regardless of their intensity and duration, cause parental and familial stress, and more stress results in less parental psychological coping [7].

Although various studies have been conducted on IEAAMs, most focus on diagnosis, screening, and treatment methods. However, the mothers of these children face many challenges, and there are few and scattered studies on the different aspects of caring for children with IEAAMs and their mothers’ experiences of care. These few studies suggest that parents face many problems due to the involvement of multiple systems [9], a low quality of life [10], worries about the ambiguous future of their children [11], and the importance of disease acceptance in taking care of their children [11, 12]. Mothers of children with IEAAMs suffer from depression, sleep disorders, social and familial relationship disturbances, and low quality of life, and have more struggles than other families [3]. Despite these overwhelming obstacles; however, these mothers can move forward, adapt to new norms and even have an optimistic outlook [11].

Due to the lack of knowledge about the experience of caring for children with IEAAMs, recent studies have suggested the need for more research in this area. For example, the UK Strategy for Rare Disease, the National Rare Disease Plan for Ireland, and many national strategies for rare diseases in Europe emphasize the importance of involving parents and families in research and using patients’ and caregivers’ opinions in policies and services that affect them. These strategies also stress the need to apply different research approaches. Among these approaches, qualitative methods can demonstrate the experience of living with a rare condition and the challenges faced by patients and caregivers [11].

Mothers are assumed to be primarily responsible for taking care of their children, while fathers are assumed to work to and provide financial support for the family [13]. Therefore, clarifying these mothers’ experiences can reveal the stories of their struggles in meeting their children’s complex social and health needs. Mothers’ stories have remained silent and are not understood by healthcare services and society. Breaking the silence and clarifying the mothers’ voices and experiences can increase insight into their challenges and help them be understood [14]. The purpose of qualitative research is to describe, understand, or explain social phenomena through a scientific and systematic process [15]. Qualitative research gives children and families the chance to share their experiences and views and gain a deeper insight into conscious care. More qualitative research is needed to support shared decision-making and planning interventions based on patients’ and families’ priorities and preferences [16]. Among these approaches, interpretive phenomenology seeks to study the personal life world or experiences as they are lived. This method provides a deeper understanding of parents’ daily life, the management, and living with a child’s disease in the context of the family, and identifies the factors that strengthen or inhibit this experience [11]. Therefore, this study was conducted to explore the experiences of mothers with a child with IEAAMs, using interpretive phenomenology to assess the challenges from their points of view.

## Methodology and methods

### Theoretical framework

Most of qualitative researches are based on the constructivism paradigm, which is also referred to as naturalism or interpretivism [17]. Naturalism emerged in opposition to the positivism movement with writers such as Weber and Kant. For naturalistic inquirers, reality is not a fixed entity but rather a construction of the individuals participating in the research [18]. According to naturalism, the proper subject matter for humanity is the human world characterized by Geist- mind, thoughts, consciousness, values, feelings, emotions, action, and purposes, which find their objectifications in languages, beliefs, arts, and institutions [19]. In this philosophy, researchers emphasize the complexity of humans, their ability to shape and create their own experiences, and the idea that truth is a composite of realities. Researchers focus on understanding the human experience as it exists. Naturalistic studies yield rich, in-depth information that can elucidate varied dimensions of a complicated phenomenon. Phenomenology, in comparison to other qualitative methods investigating a phenomenon, focuses on the meaning of lived experiences of humans and a closely related research tradition in phenomenology is hermeneutics, which uses lived experiences as a tool for better understanding the social, cultural, political, or historical context in which those experiences occur. Hermeneutic inquiry, or Heidegger’s phenomenology, almost always focuses on meaning and interpretation [18]. Heidegger’s phenomenology emphasizes that human beings interpret or attach meaning to their experiences as humans. He believed, that unlike Husserl, a bracketing approach to something in a blank way was impossible. One of the methodologists who advocated a phenomenological method mainly from the Heideggerian tradition is van Manen. His method of exploring lived experience is an interpretive, artistic approach, the foundation of which is writing and language [20]. As Max van Manen stated (p. 4), “the preferred method for human science involves description, interpretation, and self-reflective or critical analysis.“ He also stated that “interpretive phenomenology is a search for the entire life” [19]. Therefore, this study was conducted through the lens of naturalism and van Manen’s phenomenology to answer the question “what is the meaning of caring for children with inborn errors of amino acid metabolism?“ by exploring their mothers’ lived experiences.

### Participants

IEAAMs are rare conditions, and all patients in the eastern part of Iran refer to Akbar Hospital, a pediatric educational tertiary hospital in Mashhad, Khorasan Razavi province. Mothers were recruited from the outpatient clinic of the Akbar hospital for inherited metabolic diseases. They had at least one child with IEAAM that was diagnosed for more than 1 year, and were willing to participate and speak in Persian. The research was done with ethical confirmation from Mashhad University of Medical Sciences (Ethical code: IR.MUMS.REC.1399.435), and all mothers completed the informed consent after explaining the study’s purpose.

A total of nine mothers aged between 22 and 50 years old participated in this study over a 12 month period through convenience and purposeful sampling. All mothers had at least one child with IEAAM, and three of them had experienced the death of a previous child due to this disease. In one case, three children’s deaths were reported. The participants had children with different kinds of IEAAMs, including maple syrup urine disease, glutaric academia, methylmalonic acidemia, urea cycle disorder, leucine disorder, homocystinuria, propionic acidemia, and citrullinemia.

### Data collection and analysis

Data were gathered through semi-structured and in-depth interviews during the participants’ inpatient visits or their child’s hospitalization. The interviews were conducted in the hospital setting since all of the participants lived far away from the hospital. The first author performed all interviews after communicating and explaining the study objectives, obtaining written consent, and collecting demographic information including the mother’s age, education, occupation, total number of children, number of affected children, child’s gender, age, educational level, disease type, and its duration. The interview began with an introductory question, “Please describe how the child’s disease was diagnosed?“ and continued with main questions such as “Please express your feelings when you found out about the metabolic disease.“ The mean duration of interviews was 25–50 min, and they were all audio-recorded and written down word for word as soon as possible. Sampling was continued until the data saturation point, where no new themes or information appeared [18].

Thematic analysis was done using van Manen’s phenomenology approach, which includes six steps: turning to the nature of lived experience, investigating experience as we live it, reflecting on essential themes, the art of writing and rewriting, maintaining a strong and oriented relation to the phenomenon, and balancing the research by considering the parts and the whole [19]. Also, for isolating thematic statements, three wholistic, detailed, and selective approaches were used. This process was performed by a research team comprising a Ph.D. student of nursing, an advisor, and a supervisory professor who had a Nursing Ph.D.

- In the wholistic or sententious approach, the researcher considers the text as a whole and tries to understand the meaning of the text and ask “What sententious phrase may capture the fundamental meaning or main significance of the text as a whole?“ [19]. In this study, each interview’s text was read over and over to obtain a general understanding. Then, the researcher interpreted the mothers’ responses using the study objectives and questions, and the general impressions of the text were summarized in some thematic statements or sententious formulations (Appendix A).
- In the selective or highlighting approach, the researcher read the text several times and asked “What statement(s) or phrase(s) seem particularly essential or revealing about the phenomenon?“ [19]. In this study, each text was read several times, and statements that helped clarify the phenomenon were extracted (Appendix A).
- In the detailed or line-by-line approach, the researcher looks at every single sentence or sentence cluster and asks “What does it reveal about the phenomenon?“ [19]. In this study, the text was read sentence by sentence to extract the thematic statements that were related to the phenomenon (Appendix A).

To achieve the trustworthiness criteria, including credibility, dependability, confirmability, and transferability, were used [21]. In this study, the trustworthiness of data based on the four mentioned criteria was confirmed as follows:

For the credibility of data, member checking was carried out. The preliminary analysis of the data was sent to participants to find out if they felt the analysis was accurate and reflective of their experiences and to solicit additional feedback in this regard [22, 23]. Also, the coding process was continuously assessed by research team and the other colleagues who were not involved in the study but were familiar with qualitative research and thematic analysis. Spending enough time gathering data, interviewing and observing participants, and continuous involvement with them was done to increase credibility as well.

To achieve dependability, accurate assessment of data and documents was done by external supervisors step by step. Also, for reaching confirmability, some interviews, codes, and themes were checked by nursing faculty members who were familiar with qualitative analysis but were not participating in this study. Lastly, Graneheim & Lundman (2004) point out that it is up to the reader to determine the transferability of the findings to other contexts. So, to assist the reader in determining the transferability characteristics of participants, data collection, and the process of analysis are included in the study [21].

## Results

Participants were 9 mothers aged between 22 and 50 years old who had at least one child with IEAAMs, all were housewives, and three of them had experienced the death of at least one child due to the disease (Table 1).

**Table 1:** participants characteristics

Participants	Participants1	Participants2	Participants3	Participants4	Participants5	Participants6	Participants7	Participants8	Participants9
Disease	Citrolinemia	propionic acidemia	Homocystinuria	MSUD	Leucine disorder	Urea cycle disorder	Urea cycle disorder	MMA	Glutaric academia
Child gender	girl	boy	girl	girl	boy	girl	boy	girl	boy
Child’s age	3 years	19 months	16 years	10 years	15 months	16 years	3 years	17 years	3.5 years
Disease duration	2.5 years	1 year	15 years	10 years	1 years	16 years	3 years	8 years	3 years
Mother’s age	22 years	28 years	50 years	30 years	34 years	42 years	34 years	50 years	26 years
Mother’s educated	Under diploma	Software engineer	none	diploma	Bachelor of Theology	diploma	Under diploma	Under diploma	diploma
Children No.	1	1	5	3	1	4	2	3	1
No. of affected child	1	1	1 and 3 death	2	1	1	2 and 1 death	2 and 1 death	1

Thematic analysis identified 6 themes and 12 subthemes (Table 2). The main themes identified were future tied to the past, disturbed psychological health in the shadow of a lost ideal child, blaming, as a way of escaping difficulties, self-forgetting in the shadow of full-time care, passing difficulties in the duality of hope and hopelessness, and caring in a continuum of isolation-socialization.

**Table 2:** main and final themes of mothers’ experience of caring a child with IEAAMs

	Main themes	Final themes
1	Confused skein of diagnosis and treatment	Future tied to past
2	Ambiguous future capture to disease	
3	Mothers’ mental disturbance	Mentally disturbed in the shadow of lost ideal child
4	Lost ideal child	
5	Child’s rebellion, Mother’s helplessness	Rebellion and blaming, the ways of escaping difficulties
6	Suffering decrease by blaming	
7	Motherhood in the shadow of disease, fear, and self-forgetting	Self-forgetting in the shadow of full time care
8	Full time care dependent to lots of restrictions	
9	Tiny candle of hope in the duality of denial-belief	Passing difficulties in the duality of hope-hopelessness
10	Passing the storm of difficulties riding the boat of time, patience, and believes	
11	Self-wanted isolation	Caring in a continuum of isolation-socialization
12	Reliving in the duality of hiding-finding sympathy	

### Future tied to past

Mothers expressed that the physical, mental and intellectual health of their children’s future is dependent on early diagnosis and on-time treatment. If the diseases were diagnosed in the neonatal period, it would be possible to prevent most of their unpleasant consequences such as intellectual disability. However, it is hard to diagnose IEAAMs as they are rare conditions and doctors know little about them. Sometimes, doctors misdiagnose them with the most prevalent diseases such as gastroenteritis or neurological disorders and order inappropriate treatments, because of their nonspecific and obscure signs and symptoms.

*Participant 1 " When she was about 4 or 5 months old, she came down with some nasty diarrhea and vomiting that lasted for a whole month. As a result, her growth totally stalled. I started giving her protein-rich foods, but since she couldn’t process the protein properly, her plasma urea levels shot up and she got all flabby. The docs kept thinking it was just a case of viral diarrhea, but by the time we finally got her to the hospital, it was too late. She even passed out and her ammoniac levels shot up to 612, throwing her into a coma. Once she woke up, we noticed she had some intellectual issues and started having seizures.“*

Moreover, mothers and children face an ambiguous future because of the wide spectrum of these diseases. The future signs and sequels are unclear and can cause physical and mental problems, from wild to severe, or even death. Therefore, parents often do not receive clear information about the prognosis and what will happen.

*Participant 2 " Those docs couldn’t make up their minds. One said the little guy wouldn’t grow, another said he’d end up with some kind of intellectual disability, and then another just shrugged and said who knows? Turns out the disease he’s got is one of those mysterious types. They’re saying there’s a huge range of things that could happen to him - it all depends on what kind of variation he’s got. Sometimes it’ll come back again and again, sometimes it’ll get worse, and sometimes it might even get a little better.“*

### Mentally disturbed in the shadow of the lost ideal child

Mothers experienced grief and sorrow as soon as they found out their children’s diagnosis of IEAAMs. The more time that passed and the more signs and symptoms appeared, the more psychological problems mothers experienced, such as depression, and anxiety, and in some cases, they even needed to go to a psychiatrist and start treatment.

*Participant 5 " I was totally clueless about this disease at first. So, I hit up the internet and each time I looked it up, the news got worse and worse. I cried so much over it. Eventually, I found out that my little boy can’t eat anything with protein, has a super-short life expectancy, and a bunch of other stuff. It hit me like a ton of bricks when I found out that there’s no cure for it. I was depressed, and crying all the time.“*

Most mothers that accepting their child’s disease was so hard because they had a healthy child for the first months or years, and then their child’s condition changed suddenly, not only physically but also mentally and intellectually. Mothers who had planned to have healthy children and who were their mothers’ entire hope and wishes had to watch the disappearance of their children’s abilities day by day, and each day waiting for new problems to happen or new signs or symptoms to appear.

*Participant 1 “If my girl had an intellectual problem from birth, even though it woulda been tough, I reckon I coulda dealt with it better if it was congenital. But she was all good and healthy for the first six months of her life, and then she became completely disabled. That’s what makes it tough - seeing her abilities, her playin’ around, and then watching her become disabled. It’s just plain hard to take.“*

### Blaming, a way to escape from difficulties

Mothers used different ways to cope with disease problems. One of these ways was blaming others, especially healthcare professionals and their partners, to escape from their problems. Mothers blamed healthcare professionals because of the late or wrong diagnosis of the condition, and their partners for not paying enough attention to the child’s health. But, in a few cases, mothers were blamed by family members because the disease was congenital and they thought it only transferred from mother to child.

*Participant 6 " I was seriously depressed for a long time while they were trying to diagnose her disease. Her physician just kept saying her symptoms were related to her neonatal jaundice and didn’t pay much attention to what I was saying. But the symptoms were just getting worse and worse. Finally, after eight long years and like, at least 16 seizures every day, they finally diagnosed her. They only checked her ammoniac level for the first time and it was at 261! The physician became upset, but honestly, it was ineffective by then.“*

*Participant 1 " I talked to my husband and I called him out for causing our kid’s condition. She was in the hospital for a whole week in our town, and the docs said she needed more tests that they couldn’t do there, so she had to go to this pediatric hospital in Mashhad. But my husband objected and said she would become well at home.“*

*Participant 4 " My family blamed me a lot because a doctor said the disease was congenital and they thought it only transferred from the mother to the child.“* 1

### Self-forgetting in the shadow of full-time care

In families with a child with IEAAMs, the burden of care fell entirely on the mothers. They noted that their children required full-time care, which was all up to them. They were also always fearful that their child would contract another illness or infection, and that their child’s condition would worsen.

*Participant 6 " Caring for this child is a full-time job, and it feels like I always have work to do. I’m never free.“*

*Participant 8 " During the night, I check on her many times, taking her hand and assessing whether she is breathing or not. When I am sure she is fine, I thank God.“*

*Participant 2 " His disease changes constantly. I am always watching his reactions and thinking about what might happen to him. When his eyes become red or his body starts shaking, I get scared that he might get a cold or become ill. I’m always scared.“*

Because of their children’s multiple problems, mothers were fully occupied with their care and often ignored their own needs and desires. They put all their attention on their children, completely forgetting about themselves.

*Participant 2 " When I see other mothers who have independence and can go out and do their own personal things, I completely forget about myself because taking care of this child is too much. I can’t do anything for myself. “*

In addition to the time consumed for caring, there were many other restrictions that made caring more difficult, such as the cost of treatment, providing vital drugs, difficulty in accessing specialized medical centers, and the challenges of preparing and providing a special diet.

*Participant 6 " The disease costs a ton, and the insurance company doesn’t have my back. I have to pay for all her meds out of pocket because the insurance only covers a fraction of what she needs. They only cover 10–15 pills per month, but she takes 4 every day. The biggest issue for me is making sure she has her meds. I have to get them before we run out, otherwise her ammoniac level shoots up, and she gets sick with seizures.“*

### Passing difficulties in the duality of hope/hopelessness

In order to face the problems of caring for children with IEAAMs, mothers used different ways, which were sometimes completely opposite to each other. Some of them believed in a complete cure for the disease in the future, while others some denied the disease and tried to provide some hope for themselves to bear the difficulties.

*Participant 2 " I haven’t believed his disease yet. I’m still hoping he doesn’t have it and that it will be cured completely. “*

*Participant 1 " I’m hoping something happens and she gets cured.“*

On other hand, there were some mothers who lost their hope for changing their child’s condition or finding a cure for it. After losing hope, they decided to accept the disease and handle the situation with patience as time passed.

*Participant 1 " Now when I see other moms, I tell them about my diaries. It’s hard to accept a problem at first, but once you do, you’ll feel better and at least won’t feel guilty. I tell them to wait and see, things will be better with time.“*

### Caring in a continuum of isolation-socialization

Most mothers with a child with IEAAMs preferred not to communicate with others and chose isolation for multiple reasons, including the amount of time caring for the, wanting to avoid others’ judgmental looks, and also to avoid answering others’ numerous questions about the child’s condition. Therefore, they selected self-imposed isolation instead of socialization.

*Participant 2 " Since my son’s health issue, I haven’t gone to parties because people always ask if he doesn’t speak or walk, or why I don’t feed him certain foods, etc. and that’s so annoying to me. I haven’t told anyone about my son’s disease because it’s unknown, and if I try to explain it, it would take at least 1–2 hours. What people say bothers me the most.“*

However, some mothers tried to speak to others to find sympathy and relief. They found comfort in communication and therefore chose socialization instead of isolation.

*Participant 1 “I used to chat up anyone next to me, even on the bus or subway. I thought for sure everyone would sympathize with me and totally get where I was coming from. I loved getting pity from anyone who would give it to me.“*

## Discussion

The purpose of this study was to understand the meaning of caring for a child with inborn errors of amino acid metabolism for mothers. After researching these mothers’ experiences, six main themes were revealed that comprise this meaning, which include the future tied to the past, psychosis in the shadow of a lost ideal child, blaming, a way of escaping difficulties, self-forgetting in the shadow of full-time care, and passing difficulties in the duality of hope-hopelessness, and caring in a continuum of isolation-socialization.

Mothers’ experience of caring for a child with IEAAMs began with the diagnosis of the problem. However, the diagnosis of such disease was difficult, challenging, and time-consuming, making it one of the most important problems for them. Other studies have also shown that the diagnosis of IEMs in different countries often takes too much time, and most of the time, there is a delay in diagnosis. For example, Khangura et al. (2016) confirmed that the diagnosis of IEMs always accompanies doubts and reaching a final diagnosis can take time, and there are many unknowns about the prognosis and future of these children [24]. Also, Pelentsov et al. (2016) and Berglund (2010) explained that reaching the final diagnosis in rare diseases, including IEMs is difficult [25, 26]. Deuitch et al. (2021) described the diagnosis of these diseases as time-consuming and may take some years [27]. Yamaguchi et al. (2016) quoted Kubo et al. (2008) stating that the delay in diagnosis is due to healthcare professionals’ lack of knowledge about these diseases [28].

The consequences of late diagnosis in IEAAMs are many and can affect children’s health and future, subsequently affecting the mothers’ caring experience. The more delay in diagnosis, the more physical and mental sequels occur in children, disease progression increases, and it leads to inappropriate treatment [29]. This can cause anxiety, stress, and worries in parents [29]. They think there must be a diagnosis describing the condition of their children, and it is their duty to find it. They think a diagnosis can help them in coping with the complicated caring needs of their children. Parents feel that without a diagnosis, they could not prepare themselves for the changed condition of their children, and this insecurity is stressful for them [26].

Because the stress associated with IEAAMs diagnosis was an extremely traumatizing event for mothers and caring for children with these diseases had numerous stressors, most mothers experienced psychological problems. These problems are common and reported in various studies, including anxiety and stress [28, 30, 31], negative feelings, anger, denial, and depression [12], loneliness [25], physical and emotional stress, and feeling of inadequacy [32], internalization problems such as anxiety, depression, mood disorders, interpersonal challenges, low quality of life, grief, and sorrow [33, 34], and indigestion problems [33].

Our study showed that mothers tend to blame other people to alleviate some of their pains, and in some cases, they are the ones who are blamed for their child’s disease. Generally, it is mothers who blame healthcare professionals because the diagnosis process took so much time. Like our study, Eiser et al. (1995) and Garau (2016) noted that parents usually blamed the healthcare professionals for their children’s situation. They believed that the healthcare professionals’ lack of knowledge about rare diseases resulted in late diagnosis, which consequently caused physical and mental sequels for the children [35, 36]. Conversely, sometimes parents are the ones who are blamed by others or themselves [37, 38], especially mothers in genetic disorders [38]. James et al. (2006) reported that after diagnosing a rare disease in a child, parents often respond to it in the form of guilt and blame. Blaming themselves and others is a form of psychological defense against a strong feeling of helplessness [38].

On the other hand, mothers felt that most of the burden of caring for their children and managing their disease were on them, which imposed many restrictions for them. Children with IEAAMs were partially or completely dependent on parents for care and daily activities because of two main reasons. First, IEAAMs were generally diagnosed in infancy when the children had no ability to care for themselves, and secondly, these disorders affected multiple body systems which required lifelong care and treatment. This result is also reported in other studies such as Plentsov et al. [26]. In order to decrease the effects of disease, mothers sacrificed themselves for their caring role and became caregivers and therapists for their fragile and vulnerable children, even at the cost of losing themselves [39]. It is difficult for them to find a balance between work and child care, and it causes some mothers to leave their jobs or decrease their work time, and their professional aspirations lose their priority [26, 40]. Therefore, they gradually forgot about themselves to provide full-time care for their children.

Disease acceptance was difficult for mothers, and they faced it with two different approaches. Some of them were hopeful that there might be a cure for the disease, or the diagnosis might be wrong, and their children could be healthy. Some of them lost their hope, and after a while, accepted the disease and its problems. In another study, Carpenter et al. (2018) mentioned that not all parents could accept the disease and preferred that it didn’t exist, wishing they had a normal child [12]. On the other hand, some families try to increase their hope through religious beliefs and pass through the difficulties. Nematollahi (2019) revealed that parents try to care more through appreciation and communication with God [41]. Zengin (2020), also, showed that parents could cope with their problems by believing in God and life after death [9].

Another big challenge of mothers having a child with IEAAMs is the way others look at their sick child, which causes them to feel stigmatized. In studies, multiple factors were introduced as the reasons for this feeling. For example, parents thought that even a different way of feeding the child drew people’s attention and made them feel unsatisfied [33, 42]. Consequently, parents decided to decrease their relationships to avoid others’ heavy looks, frequent questions, and comparisons of their child with healthy ones. Diesen (2014) showed that the area of stigma management is a continuum in which intimacy and family isolation are its ends [42]. Parents may feel unrelated, or conversely, expand their relations as fighters and saviors until their voices are heard, and necessary services in health and social systems are gained [39]. Vegni et al. (2009) revealed that patients thought the disease was not the subject by itself, but rather continuously maintaining the difficult balance between socialization and isolation is their problem [43]. Also, Ford (2018) and Plentsov (2016) showed that most parents experienced social isolation [26, 33]. Stigma, misunderstandings, and feeling rejected by society were also common [33].

## Conclusion

This is the first qualitative study that explores mothers’ experiences of having a child with IEAAMs. Our findings demonstrate that mothers face multiple challenges in taking care of their children. They are mentally disturbed following the diagnosis of IEAAMs, a chronic and life-threatening disease that affects multiple systems, they often tried to accept it through patience, beliefs, and sometimes blaming others or themselves. We also demonstrated mothers take care of their children full-time, which results in self-wanted isolation and self-forgetfulness. These findings reveal the importance of providing psychological support for mothers. Additionally, we highlight the possibility of preventing physical and mental problems in children and increasing their quality of life by providing fast and on-time diagnosis of these diseases, which can be done through screening tests in neonates. We recommend studying other inborn errors of metabolism and planning supportive programs for both children and their mothers.

## Clinical implication

The findings of this study have revealed the experiences, challenges, and problems of mothers caring for children with inborn errors of amino acid metabolism. Therefore, healthcare professionals can use these experiences to understand the feelings of mothers and their needs in caring for children with IEAAMs, and use them in planning the care of these children, such as a shared care plan, and improve the quality of life for mothers, children, and subsequently, the whole family.

## Study limitation

This study was conducted on families referring to one center, which decreases its generalizability to an international setting, although this center is a tertiary one to which families with different cultures have referred. The authors also tried to include participants with different experiences, such as the duration of the disease and the number of affected children in families. Additionally, this study was conducted during the COVID-19 pandemic, which made it impossible to interview mothers in their homes for a more comprehensive assessment of their daily lives to prevent infection. All interviews were conducted in our center when the children were hospitalized or visited in a day clinic.

## Electronic supplementary material

Below is the link to the electronic supplementary material.

Supplementary Material 1

## Data Availability

The datasets used and/or analyzed during the current study are available from the corresponding author upon reasonable request.

## Footnotes

1 - In Persian the “congenital” term is translated as “maternal” and that’s the reason caused some people thought it is the mother’s fault.

## List of abbreviations

IEAAM: inborn errors of amino acid metabolism
IEM: Inborn Errors of Metabolism
SIDS: sudden infant death syndrome.
