# Correction: Mothers’ lived experience of caring for children with inborn errors of amino acid metabolism

**DOI:** 10.1186/s12887-023-04133-8

**Published:** 2023-06-19

**Authors:** Sara Shirdelzade, Monir Ramezani, Peyman Eshraghi, Abbas Heydari

**Affiliations:** 1 Department of Pediatrics, School of Nursing and Midwifery, Mashhad University of Medical Sciences, Mashhad, Iran; 2 Nursing and Midwifery Care Research Center, Mashhad University of Medical Sciences, Mashhad, Iran; 3 Department of Pediatric and Endocrinology, School of Medicine, Mashhad University of Medical Sciences, Mashhad, Khorasan Razavi Province Iran

**Correction:** ***BMC Pediatr*** **23** **, 285 (2023)**

10.1186/s12887-023-03946-x

Following the publication of the original article [1], the authors have identified an error in the 3rd and 4th authors’ affiliation, which they would like to correct.

The correct affiliation of the 3rd and 4th authors should be as follows:

• **The 3rd author: Peyman Eshraghi**, Associate Professor, Department of Pediatric and Endocrinology, School of Medicine, Mashhad University of Medical Sciences, Mashhad, Iran

• **The 4th author: Abbas Heydari**, Professor, Nursing and Midwifery Care Research Center, Mashhad University of Medical Sciences, Mashhad, Iran

The original article has been corrected.
