# Supplementary material for: Mothers’ lived experience of caring for children with inborn errors of amino acid metabolism
Source: BMC Pediatr. 2023 Jun 7;23:285. doi: 10.1186/s12887-023-03946-x (PMC10244838; doi:10.1186/s12887-023-03946-x)
Supplement: Supplementary file 1 — Supplementary Material 1 [file 12887_2023_3946_MOESM1_ESM.docx]

Appendix A

Examples of three approaches to isolating thematic statements

1. Wholistic approach

Here is a part of a story of a 22-year-old mother with a daughter with citrullinemia

"A healthy 6-months-old girl became ill after solid food added to her diet other than breastmilk. She developed diarrhea and became more ill day by day and after a few weeks, she went into a coma. It takes 5 tough days for the mother that her daughter came out of a coma and then suddenly she faced a disaster; her healthy daughter lost all her physical and mental abilities…"

Thematic statement extracted from this part of her story: "a mother who lost her healthy child during few weeks"

1. Selective approach

A 28-year-old mother said "I still didn't believe it. We just did MS/MS test, not a genetic test. Although, he has all the signs and symptoms of the disease; there is a possibility of not having the disease because we haven't done the genetic test yet"

Thematic statement extracted from these sentences: "Resistance to disease acceptance"

1. Detailed approach

A 30-year- old mother with a daughter with MSUD in response to the "how do you feel when you found out your daughter's disease?" after a few seconds of silence said "well, nothing. I just cried because others blamed me for her disease. [This time she silenced for several minutes]"

The themes revealed were: silence, crying, and blaming
